# The Nutrient Rich Food Price Index: a nutrition-relevant adaptation of the Laspeyres price index to track the cost of affordable nutrient density

**DOI:** 10.3389/fnut.2023.1107573

**Published:** 2023-05-19

**Authors:** Alfonso Mendoza-Velázquez, Mariano Guzmán-Rodríguez, Jonathan Lara-Arévalo, Adam Drewnowski

**Affiliations:** ^1^Dirección General de Investigación Económica, Banco de México, Mérida, México; ^2^Departamento de Economía, Universidad Popular Autónoma del Estado de Puebla (UPAEP), Puebla, México; ^3^Centro de Investigación e Inteligencia Económica, Universidad Popular Autónoma del Estado de Puebla (UPAEP), Puebla, Mexico; ^4^Center for Public Health Nutrition, University of Washington, Seattle, WA, United States

**Keywords:** Laspeyres index, nutrient rich food price index, food prices, nutrition economics, affordable nutrient density, Mexico

## Abstract

**Background:**

The Laspeyres price index is the ratio of the current cost of a market basket of commodities or food groups relative to base period prices.

**Objective:**

To develop a nutrition-relevant version of the Laspeyres price index, using market baskets based on tertiles of the nutrient rich food (NRF9.3) nutrient density metric.

**Methods:**

Nutrient composition data for 151 foods from the 2012 Mexico national health and nutrition survey (ENSANUT) were merged with food prices and price indices from the national institute of geography and statistics (INEGI). Nutrient Rich Food Index (NRF9.3) was the measure of nutrient density. May 2012 was the base period. Nutrient rich food price index (NRFPI) values were calculated for each tertile of NRF nutrient density scores for each month between June 2011 and March 2022.

**Results:**

The market basket of foods in the top tertile of NRF nutrient density scores cost more per 100 kcal and had higher NRFPI values compared to foods in the bottom tertile. Higher NRF9.3 scores were correlated with greater monthly inflation. The NRFPI for foods in the top tertile of NRF9.3 scores was marked by seasonal price spikes, and greater volatility compared to foods in the bottom tertile.

**Conclusion:**

The present adaptation of the Laspeyres Index used market baskets defined by nutrient density tertiles instead of commodity groups. This approach allows for easier tracking of the cost of nutrient dense foods and healthful diets across geographic regions and over time. Applied to Mexico food prices prior to and during the Covid-19 pandemic, the NRFPI was sensitive to time trends, seasonality, and price fluctuations. The new tool may be useful in monitoring the rising cost of healthy foods worldwide.

## 1. Introduction

Assessing the rising cost of healthy foods can be done in a number of ways. The classic Laspeyres price index is computed as the ratio of the current cost of a market basket of commodities relative to the cost of the same commodities at base-period prices (1, 2). The rising cost of commodities is routinely monitored by the Food and Agriculture Organization (FAO) (3), World Bank (WB) (4), the United States Department of Agriculture (USDA) (5), and other domestic and international agencies. Based on those reports, food price inflation has been reaching record levels. However, the estimated costs are typically computed per commodity weight or volume (6, 7). Many of the commodities tracked by price indices differ greatly in energy content and in nutritional value (8, 9).

The present goal was to develop a nutrition-relevant version of the Laspeyres index to track affordable nutrient density globally, with a special focus on the low- and middle-income countries (LMIC). Measuring the affordability of healthy foods is the main focus of the 2022 FAO’s State of Food Security and Nutrition in the World report (10). A number of studies have explored the nature of least-cost healthy diets (11, 12) using a variety of affordability indicators.

The present innovation was to replace the market basket of commodities in the Laspeyres price index with market baskets based on tertiles of the nutrient rich food (NRF_9.3_) nutrient density metric. The Nutrient Rich Food Index is a well-established quantitative tool to assess nutrient density of foods (13, 14). It is an across-the-board score, meaning that the same nutrient standards are applied across all food groups (15). As NRF9.3 scores are split into tertiles, foods in the top or in the bottom tertile of nutrient density can come from multiple food groups (13).

Developing new metrics to assess the monetary cost of essential nutrients across different LMIC can aid international agencies to compute the cost of healthy diets. The present proof-of-concept analyses were based on nutrient density tertiles for 151 foods from the national health and nutrition survey (ENSANUT) in Mexico (16), linked with monthly average food prices from Mexico federal agencies for the period 2012 to March 2022 (17).

## 2. Materials and methods

### 2.1. Nutrient composition database

Energy and nutrient composition data for frequently eaten foods in Mexico (*n* = 151) came from the 2012 round of national health and nutrition survey. The list included corn and flour tortillas; grains; meats and fish; snacks (including sweets and nuts); milk and dairy; beans and eggs; fruits and vegetables; sugars, fats and oils; and sugar sweetened beverages (16).

### 2.2. Nutrient density evaluation

The Nutrient Rich Food Index has two components: NR9 and LIM3. The positive NR9 component is the sum of percent daily values for nine nutrients to encourage $(Nut\_inc_{i}$ ): protein, fiber, vitamin C, vitamin A, calcium, iron, potassium, magnesium, and vitamin D. The negative component (LIM_3_) is based on the sum of percent daily values for saturated fat, added sugar, and sodium (14). Percent daily values are capped at 100%. The NRF_9.3_ score is calculated as follows:


$$\begin{array}{l} NR9=(\frac{protein}{50g}+\frac{fiber}{25g}+\frac{vit\;A}{800RAE}+\frac{vit\;C}{90mg}+\frac{vit\;E}{9mg}\\ +\frac{Ca}{1000mg}+\frac{Fe}{18mg}+\frac{K}{3500mg}+\frac{Mg}{310mg}) \end{array}$$

$$LIM3=\left(\frac{sat\;fat\;}{20\;g}+\frac{addedsugar\;}{50\;g}...+\frac{sodium}{2000mg}\right)$$

$$NRF_{9.3}=\left[\sum_{i=1}^{9}\frac{Nut\_inc_{i}}{\left(DV_{i}\right)}-\sum_{j=1}^{3}\frac{Nut\_Lim_{j}\;}{\left(MRV_{j}\right)}\right]x\left(100/energ\_dens\right)$$

Nutrient standards were based on the Mexico national institute of public health (INSP) and the Codex Alimentarius (18). The reference daily values (*DV_i_*) were protein (50 g), fiber (25 g), vitamin A (800 RAE), vitamin C (90 mg), vitamin E (9 mg), Ca (1,000 mg), Fe (18 mg), K (3,500 mg), and Mg (310 mg). The maximum recommended values (*MRV_j_*) were saturated fat (20 g), added sugar (50 g) and sodium (2,000 mg). The NRF_9.3_ index scores, split into tertiles of nutrient density, were then used to calculate the nutrient rich food price index (NRFPI).

### 2.3. The Nutrient Rich Food Price Index (NRFPI_9.3, t_)

Average prices (per kg or L) for the 151 foods, coinciding with the ENSANUT survey period and denominated in Mexican Pesos (MXN), were obtained from the National Institute of Geography and Statistics (INEGI). Data collection procedures have been published (19). Food prices were corrected for preparation and waste and were expressed in MXN/100 g and MXN/100 kcal, edible portion. Monthly inflation rates for each food, also available from INEGI, were used to establish prices between June 2012 and March 2022. The annualized monthly inflation rates $\left(\pi_{t}\right)$ were calculated using average food prices $\left(p_{t}\right)$ in a given month as follows $\pi_{t}=\left(\frac{p_{t}}{p_{t-12}}\right)-1$ , where $p_{t-12}$ is the food price twelve months before. Affordability was the ratio of *NRF_9.3_* score points and cost (MXN/100 kcal):


$$Affordability=\frac{NRF9.3}{\left(\frac{MXN\$}{100\;kcal}\right)}$$

The present adaptation of the Laspeyres price index (1, 2) used market baskets based on nutrient density tertiles instead of commodities. The *NRFPI_9.3,t_* price index score was computed as the average of *m* food price indexes weighted by the respective food NRF_9.3_ score, which is held fixed over time. Using the NRF_9.3_ as the main nutrient density indicator and metric of scale (15), the *NRFPI_9.3,t_* attached projected food prices (per 100 g or 100 kcal) to individual *NRF_9.3_* scores starting in June 2011. The estimation of average food prices for the whole sample period employed the monthly growth of individual price indexes to project every price at time t. The present version or the NRFPI was related to the NRF9.3 nutrient density score and can be called NRFPI_9.3,t_. The NRF9.3 score was the time-invariant weight attached to individual food prices as follows:


$$NRFPI_{9.3,t}=\frac{P_{1,t}•NRF_{9.3,1}+•••+P_{m,t}•NRF_{9.3,m}}{P_{1,0}•NRF_{9.3,1}+•••+P_{m,0}•NRF_{9.3,m}}$$

where $P_{i,t}$ are the average prices of foods i=1,...,m in time t . The nutrient scores for each food i , e.g., *NRF_9.3,1_*,…, *NRF_9.3,m_*, described above, serve as time-invariant weights for each price index. The *NRFPI_9.3,t_* was constructed using variable prices and fixed nutrient scores as weights. This *NRFPI_9.3,t_* keeps nutrient density constant while allowing prices to vary over time. The base month *t*=0, where prices and nutrient scores are fixed, was May 2012, the end month of ENSANUT 2012 data collection.

### 2.4. Econometric and statistical analyses

Descriptive analyses examined the association between nutrient density and nutrient cost. First, we examined energy density, food prices and affordability by NRF*_9.3_* tertiles. Second, the relation between *NRF_9.3_* scores, energy density, and prices per 100 g and 100 kcal was examined using scatter plots. Next, the time series properties of food prices were analyzed to assess dynamics both on MXN/100 kcal and MXN/100 g over the sample period. Mean food prices in May 2012 constituted the base period. A battery of tests assessed linear trends and seasonality using dummy variables, *t*-tests, analysis of variance and *F*-tests. The association between price levels and inflation with the degree of nutrient quality is tested using Wald tests.

This study used intercept dummy variables to test for linear trends and seasonality of *NRFPI_9.3,t_* monthly nutrient costs and annualized monthly rates of inflation. The model adds time trends that were jointly tested employing individual *t-tests* and Wald *tests* in the parameter estimates of the following regression:


$$NRFPI_{9.3,t}=\beta_{0}+\sum_{m=1}^{11}\beta_{m}D_{m}+\theta •t+u_{t}$$

Where $NRFPI_{9.3,t}$ are the nutrient price indexes of each tertile group per 100 kcal (MXN$ /100 kcal) or 100 g (MXN$ / 100 g), respectively; $D_{m}$ are the *m=[1,…,11]* seasonal dummy variables with one for every month and *0* otherwise. The linear time-trend variable is t with a gradient parameter (*θ*). The average shift impact of each seasonal month to the conditional mean is captured individually by $\beta_{m}$ , while θ is the average impact of each passing month on $NRFPI_{9.3,t}$ scores. Linear OLS estimations methods assume that $u_{t}\sim i.i.N.\left(0,\sigma^{2}\right).$ As in previous studies (20), these tests are based on robust standard errors to account for potential violations of residuals' independence.

## 3. Results

### 3.1. Tertiles of nutrient density

Table 1 shows energy density (kcal/100 g), NRF_9.3_ values, food prices (per 100 kcal and 100 g) and affordability metrics by NRF9.3 tertiles. Foods in the bottom NRF9.3 tertile were characterized by the highest energy density (287.41 kcal/100g), lowest cost (MXN$ 3.10/100 kcal) and low nutrient density to price ratio (−5.52). Foods in the top tertile of NRF 9.3 scores had lower energy density and were more expensive (MXN$ 5.40/100 kcal) but had a better nutrient density to price ratio (27.21) A similar pattern is observed with nutrient affordability measured per 100 g.

**Table 1:** Energy density, nutrient density (NRF_9.3_), food prices (MXN$ per 100 kcal) and affordability by NRF_9.3_ tertiles.

NRF9.3	Energy density (kcal/100 g)	Nutrient density NRF_9.3_	MXN$/100 kcal	MXN$/100 g	Affordability	
	Mean (SE)	Mean (SE)	Mean (SE)	Mean (SE)	NRF9.3/MXN$/100 kcal	NRF9.3/MXN$ /100 g
Tertile 1	287.41 (29.67)	−17.11 (3.04)	3.10 (0.36)	5.47 (0.48)	−5.52	−3.13
Tertile 2	202.12 (21.73)	27.92 (1.67)	2.94 (0.62)	4.47 (0.92)	9.50	6.25
Tertile 3	113.43 (20.93)	146.94 (12.35)	5.40 (0.59)	3.45 (0.51)	27.21	42.59
All items	201.56 (16.21)	51.82 (7.03)	3.81 (0.33)	4.47 (0.39)	13.60	11.59

### 3.2. Nutrient Rich Food Price Index time trends by NRF_9.3_ tertiles

**Figure 1:**
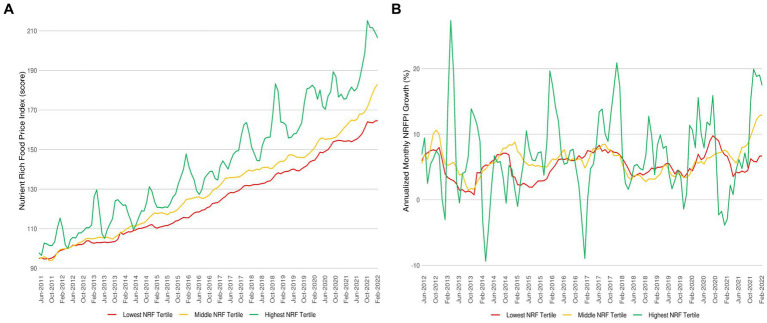
Nutrient rich food price index (NRFPI) per quality tertiles and NRFPI monthly inflation rates per quality tertiles. **(A)** NRFPI per 100 kcal per NRF_9.3_ quality tertiles panels. **(B)** Annualized NRFPI monthly inflation rates per nutrient rich food (NRF_9.3_) quality tertiles.

Figure 1A shows the time series behavior of the nutrient rich food price index (NRFPI_9.3,t_) between June 2011 and March 2022. For foods in the top tertile of NRF_9.3_ scores, the NRFPI index rose from 100 base period points to 206.32 score points in March 2022 (an annualized average monthly increase of 7.13%). For foods in the middle tertile of NRF scores, the NRFPI rose from 100 points to 183.07 points (a monthly increase of 6.21%); while for the foods in the bottom tertile of the NRFPI rose from 100 to 164.56 points (a monthly increase of 5.21%). Figure 1A further shows that foods in the top tertile of NRF_9.3_ scores not only cost more but also showed more price spikes and more price volatility than foods in the bottom tertile. Foods in the top tertile of nutrient density scores included fresh produce.

**Figure 2:**
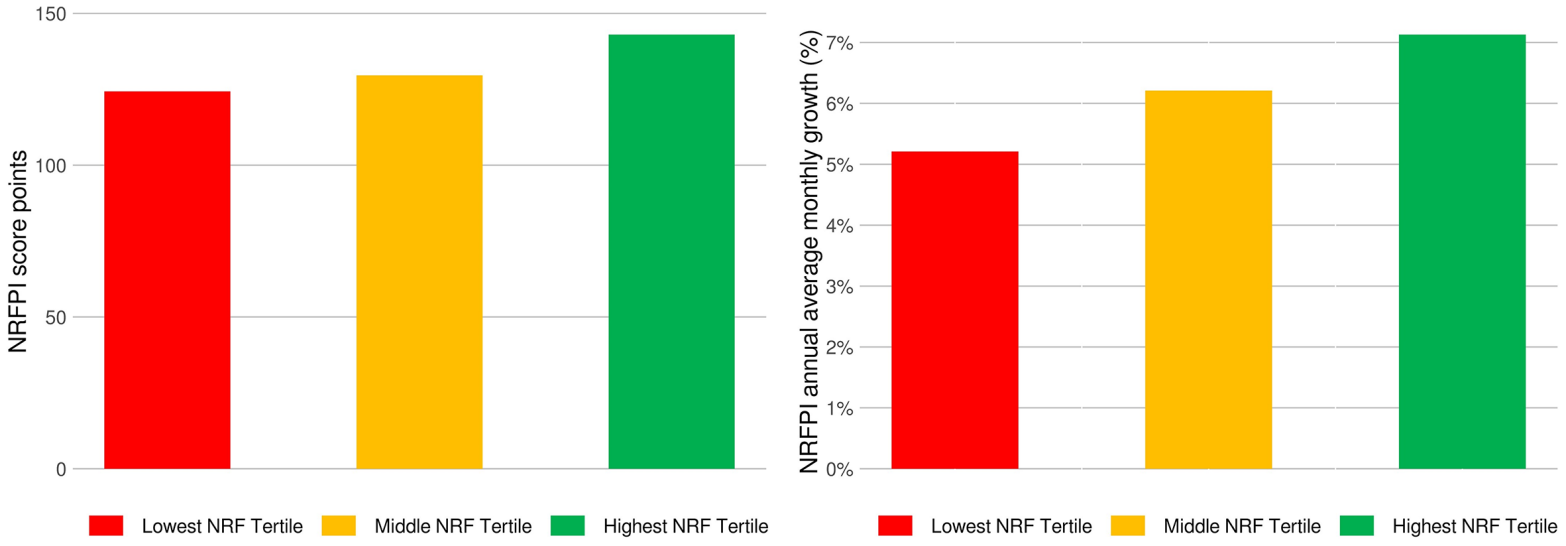
Average nutrient rich food price index (NRFPI) scores and inflation rates from June 2011 to March 2022.

While the price gap (% difference) between NRFPI price indexes was set to zero in May 2012, by March 2022 the cost gap grew to 12.70% compared to mid NRF9.3 tertiles and by 25.38% compared to low NRF9.3 tertiles. Figure 1B shows annualized monthly inflation rates by tertiles of NRF_9.3_ scores. Inflation rates were higher for the more nutrient-rich foods as compared to foods in the bottom NRF tertile. What is more, price increments for the most nutrient rich foods were highly erratic. Figure 1B also shows a significant increase in costs for all foods starting in 2021, likely associated with the global increases in food prices from mid-2021. Figure 2 shows NRFPI average price levels and annualized monthly inflation rates by tertiles of NRF_9.3_ nutrient density.

**Figure 3:**
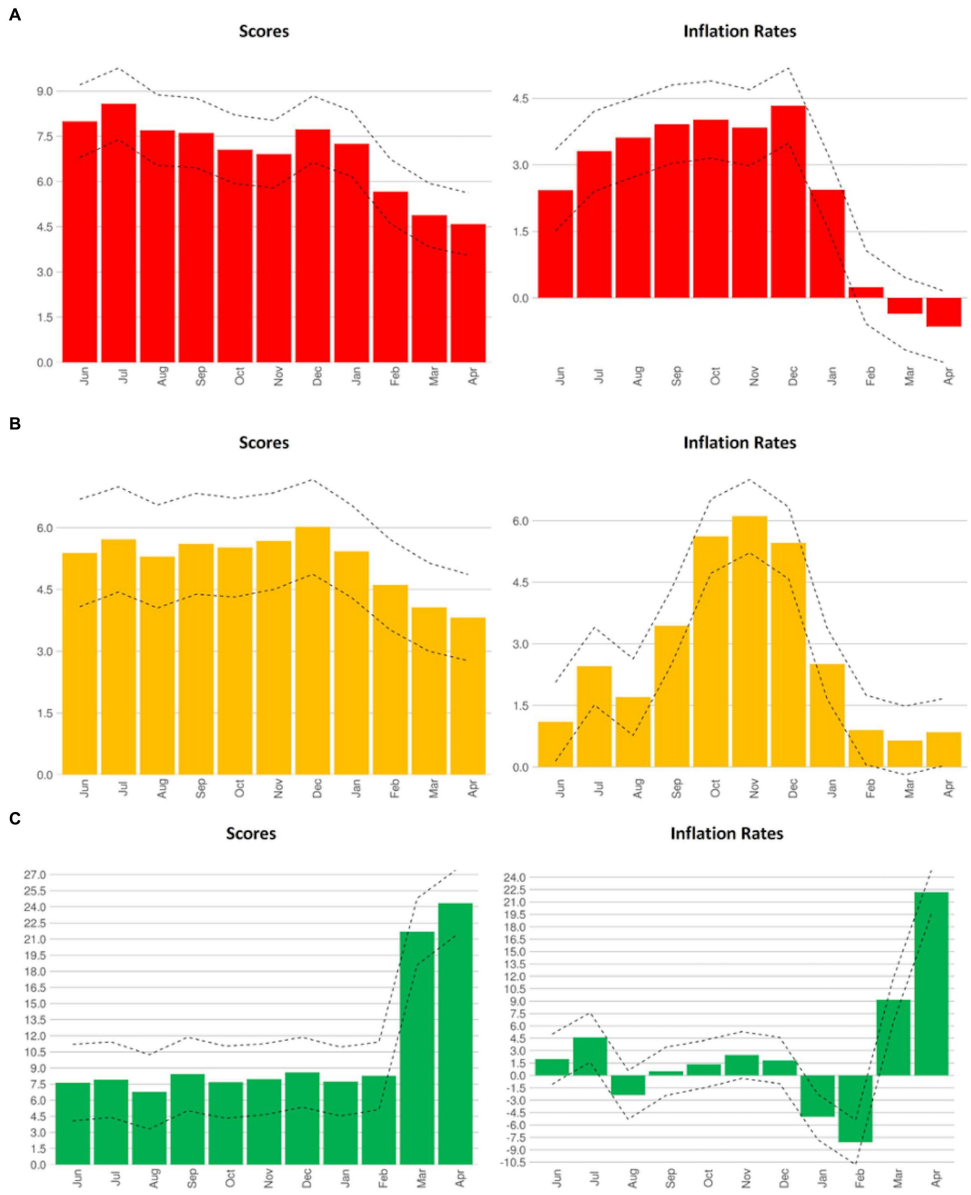
Seasonality average monthly impacts on nutrient rich food price index (NRFPI) (scores) and monthly inflation rates (%), from May 2012 to March 2022. **(A)** Low nutrient rich food (NRF_9.3_) tertile. **(B)** Mid NRF_9.3_ tertile. **(C)** High NRF_9.3_ tertile.

Table 2 shows mean difference in NRFPI, inflation rates and seasonality trends by tertiles of NRF_9.3_. Higher-scoring foods were associated with higher inflation rates. The trend gradient (θ) also rose from 0.58 for foods in the bottom tertile of NRF_9.3_ to 0.64 for the middle tertile and 0.84 for the top tertile. The historical monthly price increases were higher for the more nutrient rich foods. Finally, seasonality tests based on dummy variables for NRFPI levels showed differential increases in monthly costs by NRF_9.3_ tertiles (see Figure 3). The analysis employing annualized monthly inflation rates also confirms the strong presence of seasonality effects. While the source of seasonality effects still needs to be determined, the monthly price and inflation variations may be related to weather effects, harvesting seasons, and local and international trade patterns (20). The pattern of prices and inflation rates for low and middle NRF9.3 tertiles reveals a more stable seasonal variation of nutrient prices compared to the irregular seasonal behavior of prices observed in highly nutritious foods. The relatively high spikes of seasonality in the prices of highly nutrient foods in particular months suggest potential trade anomalies and may have an impact on diet quality.

**Table 2:** Nutrient rich food price index (NRFPI_9.3,t_) & monthly growth of nutrient costs (descriptive statistics), trends and seasonality, June 2011—March 2022.

NRF_9.3_ tertiles	NRFPI					Inflation rates (%)					Trends & seasonality of NRFPI	
Level	Mean	RSE^†^	VC^‡^	Min.	Max.	Mean	RSE^†^	VC^‡^	Min.	Max.	*θ*	Wald
Bottom	127.1	1.75	0.15	100.6	164.6	5.22	0.18	0.39	0.75	9.77	0.58^*^	6.66^*^
Middle	132.9	1.96	0.16	100.5	183.1	5.98	0.20	0.37	1.48	12.95	0.64^*^	2.11^*^
Top	147.0	2.62	0.19	104.5	215.2	6.86	0.58	0.93	–9.4	27.32	0.84^*^	1.76^*^
All items	147.3	2.62	0.19	104.4	215.0	6.90	0.58	0.91	–9.2	27.13	0.84^*^	1.74^*^

*Significance at 1% levels. ^†^RSE: robust standard errors to autocorrelation and heteroscedasticity. ^‡^VC: variation coefficient.

### 3.3. NRFPI time trends during the COVID-19 pandemic

Table 3 assesses the behavior of NRFPI scores and annualized inflation rates of foods with different nutritional values during the COVID-19 pandemic. The most significant variation of prices and inflation was observed in the last year of the sample, from March 2021 to 2022, when foods with the highest nutrient quality changed from an average score of 176.16 score points in 2021 to 207.50 points in 2022, an annual growth of 17.79%. The prices of foods in the low and mid-levels of nutrition experienced a lower rise: 6.69% and 12.96%, respectively, during the same period. The most nutrient rich foods did not only cost more per 100 kcal but also experienced a higher inflation growth at a time of health, social and economic crisis.

**Table 3:** Nutrient rich food price index (NRFPI) behavior during the COVID-19 pandemic.

	Jun 2011-Mar 2022				NRFPI (score)			NRFPI annual growth (%)		
Nutrient	NRFPI (score)		Inflation (%)		2020	2021	2022	2020	2021	2022
Density	Mean	SE	Mean	SE						
All	147.28	2.62	5.02	0.58	181.02	176.16	207.50	10.57	−2.69	17.79
Low	127.10	1.75	5.22	0.18	144.63	154.23	164.56	4.41	6.64	6.69
Mid	132.90	1.96	5.98	0.20	151.00	162.07	183.07	4.87	7.34	12.96
High	147.04	2.62	6.86	0.58	181.03	175.73	206.32	10.65	−2.92	17.41

## 4. Discussion

The present adaptation of the Laspeyres index (1, 2), matches market baskets defined by constant levels of nutrient density per 100 kcal with individual time-varying food prices (15). As far as we know, this is a novel way to allow the tracking of the cost of priority nutrients derived from multiple food groups over time. This new and nutrition relevant version of the Laspeyres index can be used to track the cost of healthy foods, with a special focus on the LMIC.

Food price indexes maintained by the FAO, the World Bank, and other national and international agencies are invaluable tools of public health policy (21). The US bureau of labor statistics (BLS) consumer price index (CPI) is a measure of change over time for a market basket of consumer goods, including food (22). For the most part, these highly important price or inflation indexes are based on the weight or volume of commodities or foods. Distinct from the CPI, the NRFPI tracks the cost a market basket of essential nutrients as opposed to foods. The quest to find an appropriate tool to measure affordable nutrient density more directly has now run into a flexible, helpful metric to track the prices and inflation of nutrients, with extensive applicability in LMIC and developed countries.

The present focus on nutrients and micronutrients is directly relevant to the concept of affordable nutrient density, as promoted by FAO. Across different LMIC, the same nutrients can come from diverse food groups. Whereas in the United States dairy is the main source of calcium by far (23), in Vietnam it is not (24). Whereas in Brazil the main food sources of iron are meats, poultry, and seafood, the main sources of iron in Mexico are fortified cereals and meats. Merging food composition databases with national food prices can help identify foods that are both affordable and nutrient rich (15, 25).

The new NRFPI is an instrument that incorporates three fundamental elements in a single score: nutrient density of foods, food prices, and time trends. Interestingly, foods in the top tertile of nutrient density showed not only the highest prices but were characterized by price spikes, variability, and seasonality. The observed seasonality effects suggest that the higher cost of nutrient rich foods (meat, fish, dairy, vegetables, and fruit) was associated with the local cycles of production and harvesting in Mexico. By contrast, foods in the lowest tertile of nutrient density (many of them processed and ultra-processed) were less expensive and were less affected by inflation. Those findings have implications for public health food policy in Mexico and likely elsewhere.

By not being tied to any commodity or food group, the NRFPI can be applied across different countries. It could become the metric for tracking affordable nutrient density across LMIC with respect to a base period. The present analyses used the NRF_9.3_ index as the measure of nutrient density; however, the same concept can be applied to other NRF indices in the Nutrient Rich Foods family of scores. The NRFPI can also be used to monitor the rising cost of a market basket of nutrients at the regional level within the same country, and for the analysis of affordable nutrient density across LMIC.

Primary nutrient sources depend on the population's eating habits. An additional property of the new NRFPI consists of its ability to incorporate specific eating habits in specific populations. Food prices alone provide a very partial view of the evolution of nutrient costs. The NRFPI tracks the cost and affordability of nutrients from all the foods in a given diet. Such flexibility allows tracking specific food sources of nutrients guided by the norm and patterns of consumption, particular to the population of interest.

A prerequisite to successfully implement the NRFPI to other LMICs is the availability of monthly time series data on food prices or individual food price indexes. National food price databases are not always readily available, even in high-income countries. Ideally, analysts should also have access to information on market retail prices, although unit prices derived from income and expenditure surveys could also be employed to implement nutrient or calorie variations of NRFPI methods. Finally, the time series analysis of trends and seasonality could benefit from new data that would allow analysts to identify the sources of monthly price variations, including agricultural, trade, and transportation costs within a given food system.

## 5. Conclusion

The new NRFPI, a nutrition relevant adaptation of the classic Laspeyres index, uses market baskets defined by nutrient density in place of the usual commodity groups. Foods are assigned into nutrient density tertiles based on scores of the well-established Nutrient Rich Food Index, which is based on nine nutrients to encourage and three nutrients to limit. Applying the NRFPI metric to food prices in Mexico showed rising costs and higher inflation rates for foods in the top tertile of NRF9.3 scores. These findings have implications for public health policy. Measuring affordable nutrient density requires new tools that monitor the rising cost of healthy foods across regions and over time. The NRFPI may be the right tool to assess the rising cost of nutrient rich foods among LMIC.

## Data availability statement

Publicly available datasets were analyzed in this study. This data can be found here: https://www.inegi.org.mx/app/indicesdeprecios/Estructura.aspx?idEstructura=112001300040&329, https://ensanut.insp.mx/encuestas/ensanut2012/index.php.

## Author contributions

AM-V conceptualized the study. MG-R and JL-A prepared the data for analyzes. MG-R and AM-V worked on the economic analysis. JL-A worked on the nutritional analysis. AM-V, AD, and JL-A drafted the manuscript. All authors contributed to the article and approved the submitted version.

## Funding

At the time of writing, AM-V received funding from the Comexus-Fulbright-García-Robles Program for Visiting Research Scholars and JL-A received funding from the Fulbright Foreign Student Program.

## Conflict of interest

AD is the original developer of the Naturally Nutrient Rich and the Nutrient Rich Food (NRF) indices. That work was supported at the time by the Nutrient Rich Coalition whose members were The Beef Checkoff Program through the National Cattlemen’s Beef Association, California Avocado Commission, California Kiwifruit, California Strawberry Commission, Egg Nutrition Center, Florida Department of Citrus, Grain Foods Foundation, National Dairy Council, National Pork Board, United States Potato Board, Wheat Foods Council, and Wild Blueberry Association of North America. AD has received grants, contracts, and honoraria from entities both public and private with an interest in nutrient density metrics and nutrient profiling of foods.

The remaining authors declare that the research was conducted in the absence of any commercial or financial relationships that could be construed as a potential conflict of interest.

## Publisher’s note

All claims expressed in this article are solely those of the authors and do not necessarily represent those of their affiliated organizations, or those of the publisher, the editors and the reviewers. Any product that may be evaluated in this article, or claim that may be made by its manufacturer, is not guaranteed or endorsed by the publisher.
